# Human Thiel-Embalmed Cadaveric Aortic Model with Perfusion for Endovascular Intervention Training and Medical Device Evaluation

**DOI:** 10.1007/s00270-017-1643-z

**Published:** 2017-04-27

**Authors:** Helen McLeod, Ben F. Cox, James Robertson, Robyn Duncan, Shona Matthew, Raj Bhat, Avril Barclay, J. Anwar, Tracey Wilkinson, Andreas Melzer, J. Graeme Houston

**Affiliations:** 1 Department of Metabolic and Clinical Medicine, Ninewells Hospital and Medical School, University of Dundee, Mailbox 1, Dundee, DD1 9SY UK; 2 Institute for Medical Science and Technology, University of Dundee, Dundee, UK; 3 Centre for Anatomy and Human Identification, University of Dundee, Dundee, UK; 4 Department of Clinical Radiology, NHS Tayside, Dundee, UK

**Keywords:** Cadaveric perfusion, Endovascular training, Endovascular aortic aneurysm repair (EVAR)

## Abstract

**Purpose:**

The purpose of this investigation was to evaluate human Thiel-embalmed cadavers with the addition of extracorporeal driven ante-grade pulsatile flow in the aorta as a model for simulation training in interventional techniques and endovascular device testing.

**Materials and Methods:**

Three human cadavers embalmed according to the method of Thiel were selected. Extracorporeal pulsatile ante-grade flow of 2.5 L per min was delivered directly into the aorta of the cadavers via a surgically placed connection. During perfusion, aortic pressure and temperature were recorded and optimized for physiologically similar parameters. Pre- and post-procedure CT imaging was conducted to plan and follow up thoracic and abdominal endovascular aortic repair as it would be in a clinical scenario. Thoracic endovascular aortic repair (TEVAR) and endovascular abdominal repair (EVAR) procedures were conducted in simulation of a clinical case, under fluoroscopic guidance with a multidisciplinary team present.

**Results:**

The Thiel cadaveric aortic perfusion model provided pulsatile ante-grade flow, with pressure and temperature, sufficient to conduct a realistic simulation of TEVAR and EVAR procedures. Fluoroscopic imaging provided guidance during the intervention. Pre- and post-procedure CT imaging facilitated planning and follow-up evaluation of the procedure.

**Conclusion:**

The human Thiel-embalmed cadavers with the addition of extracorporeal flow within the aorta offer an anatomically appropriate, physiologically similar robust model to simulate aortic endovascular procedures, with potential applications in interventional radiology training and medical device testing as a pre-clinical model.

**Electronic supplementary material:**

The online version of this article (doi:10.1007/s00270-017-1643-z) contains supplementary material, which is available to authorized users.

## Introduction

The need for training of the operator through clinical simulation within the field of interventional radiology is widely recognized [1, 2]. Despite dissimilar anatomy, ethical issues and high cost, animal models continue to provide a high-fidelity training model, with blood flow, percutaneous access and physiological function. Alternatively, human cadaveric models provide more relevant anatomy and anatomical variation in which, for the purposes of aortic endovascular procedures, the human cadaveric aorta in particular has no accurate alternative animal model in terms of vessel geometry, calibre and physiological conditions [3, 4].

Unlike other cadaveric preservation methods, Thiel [5, 6]-embalmed cadavers retain flexibility, colour, tone, have extended durability, negligible infection hazard and, most importantly for an interventional training model, vascular patency. The application of human Thiel-embalmed cadavers in medical research and teaching is increasingly recognized, due to these beneficial properties [7]. Research and development into the introduction of function to fresh human cadaveric models was initiated by Garrett [8], who first described the addition of flow into the blood vessels of fresh frozen cadavers, facilitating fluoroscopic imaging and establishing human cadavers as a model for endovascular training. Garrett’s cadaveric models were not without limitations. Aside from the short shelf life, the biological hazard and increased costs associated with fresh cadavers, the short arterial circuits with low flow rates, prevented peripheral access and therefore constrained their application as a training tool for full clinical simulation and endovascular percutaneous approach. Despite these limitations, the potential benefits of using human cadavers for interventional radiology training were established by this seminal work.

A more recent study introduced the use of Thiel-embalmed cadavers with added function for interventional training [9]. This work demonstrated the successful retrograde filling of the Thiel-embalmed cadaveric aorta to conduct a vascular intervention (TAVI). This study was limited as the cadaveric model was filled but lacked ante-grade pulsatile flow within the arterial system.

This technical note describes a flexible, robust and functional Thiel human cadaveric model with extracorporeal driven ante-grade pulsatile aortic flow, demonstrating the potential of this model in endovascular surgical training, and pre-clinical medical device evaluation.

## Materials and Methods

Three human cadavers were preserved using a previously described technique by Thiel. A 2-cm incision was made directly percutaneously into the apex of the heart, and a size 8 endotracheal tube (Portex, Smith Medical, USA) was inserted into the ascending aorta where the balloon was inflated to secure it.

### Perfusion

Extracorporeal flow was introduced directly into the cadaver vasculature via the connecting tube linking the aorta and the heart lung bypass machine (HL30 Maquet, Germany) with a modified master pump in order to recreate a pulsatile-type flow at 60 beats per minute. The master pump was prepared with 6 m of 13-mm-diameter silicone–platinum-coated tubing (Silex, UK), delivering a flow rate of approximately 2.5 L per min as the standard setting.

### Temperature Assessment

Endovascular devices with thermal memory require the temperature within the aortic lumen to be within 35–39 degrees centigrade. To achieve this temperature at the deployment site, the reservoir was heated to 60 degrees centigrade prior to perfusion and temperatures were continuously measured and recorded (TS2, Optocon, Germany). Fibre-optic thermocouple (Fotemp-4, Optocon, Germany) was located within the circuit and temperatures during perfusion recorded.

### Pressure Measurement

A continuous invasive aortic pressure measurement was taken post-intervention in two of the cadavers at three separate sites: extracorporeal circuit descending thoracic aorta and abdominal aorta. An arterial catheter (Careflow, BD, USA) was inserted into each site and attached to a pressure transducer (Standard, Baxter, UK) using a standard giving set (Protect-A-Line 2, Vygon, France) with a high-pressure bag of saline prepared to retain line patency (0.9% Saline, Baxter, UK). The pressure transducer was connected to a haemodynamic monitoring system (M3046A, HP, Germany) to provide a continuous pressure measurement (Table 3).

### Multi-Modal Imaging

The following standard parameters were used throughout interventions.

### Pre-intervention Planning

Arterial land markings for abdominal aortic stent grafting and sizing of the aorta to select appropriate device were conducted by an interventional radiologist, using the CT data, allowing the appropriate device to be selected. Secondly, nearby vessels were assessed for arterial disease to determine risks, e.g. great vessel or renal artery disease. Finally, the access vessels were assessed for lumen tortuosity and diameter to allow “safe” device delivery, deployment and delivery system retrieval.

### Intervention

The simulation was designed as a multidisciplinary clinical scenario with consultant radiologist, nurse and radiographer present.

All devices were manufactured by Captivia Delivery Systems, Medtronic Inc., USA. One cadaver had two procedures, a thoracic stent graft followed by an abdominal stent graft, bifurcating to the left and right iliac arteries of the cadaver.

**Fig. 1:**
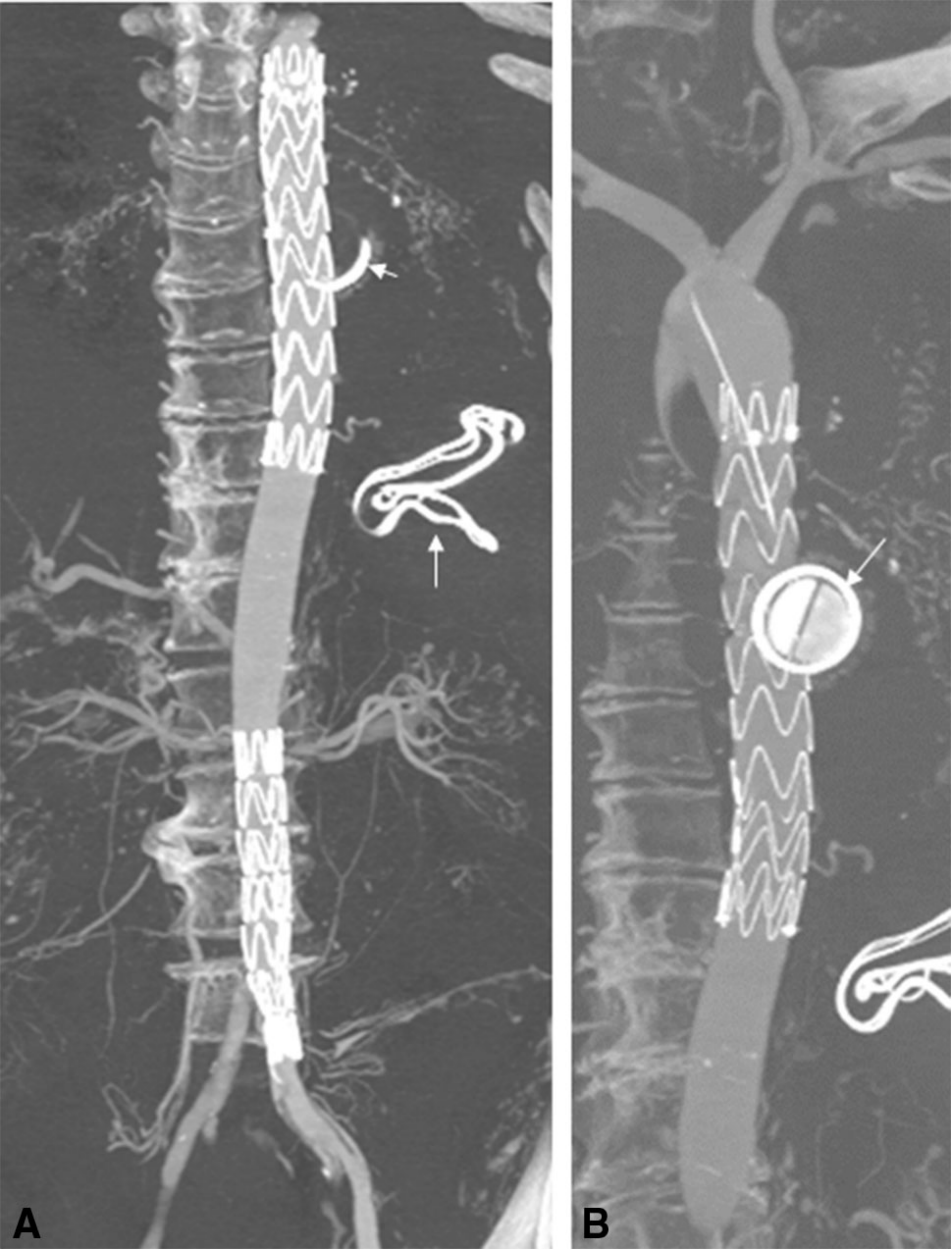
Post-procedure CT scan. CT angiogram (Table 1), MIP 3D reconstruction, allowing vessel and device evaluation (*arrows* point at post-mortem devices unrelated to this work). **A** Thoracic stent and abdominal stent conducted under fluoroscopic guidance, **B** thoracic stent in situ

**Fig. 2:**
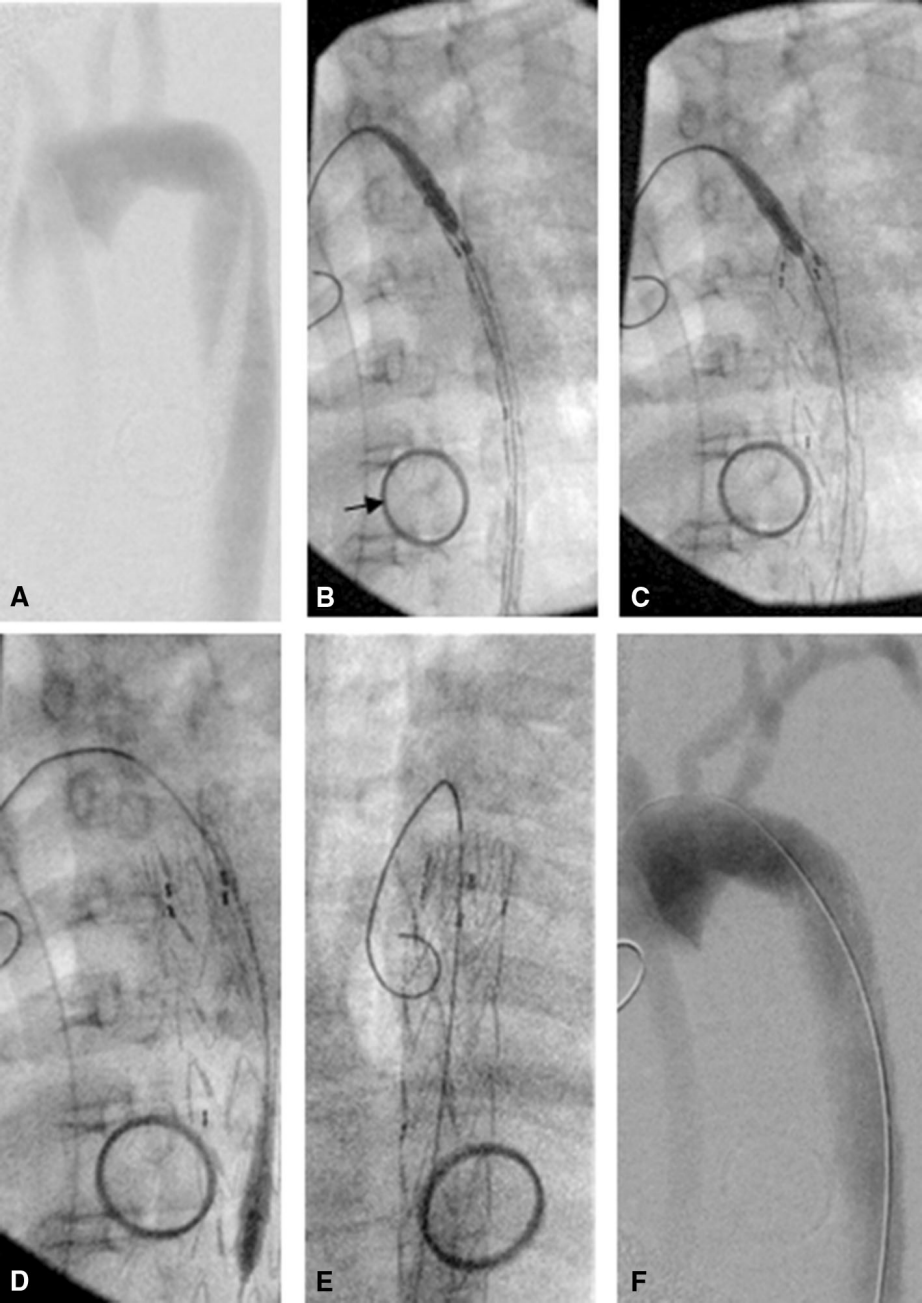
Valiant thoracic stent graft Captivia Delivery System intervention to the thoracic aorta of a Thiel-embalmed human cadaver. **A** Digital subtraction angiography cadaveric aorta with Captivia catheter located in the descending aorta. **B** Fluoroscopic-guided catheter advanced to position in cadaveric aortic arch. *Arrows* points to ring and swab placed during post-mortem training which was not related to this procedure. **C** Stent graft deployed in the thoracic aorta. **D** Catheter undocked and withdrawn. **E** Guide wire remains in device to allow angiographic catheter to be placed in aorta. **F** Post-procedure digital subtraction angiography demonstrating patent aorta

**Fig. 3:**
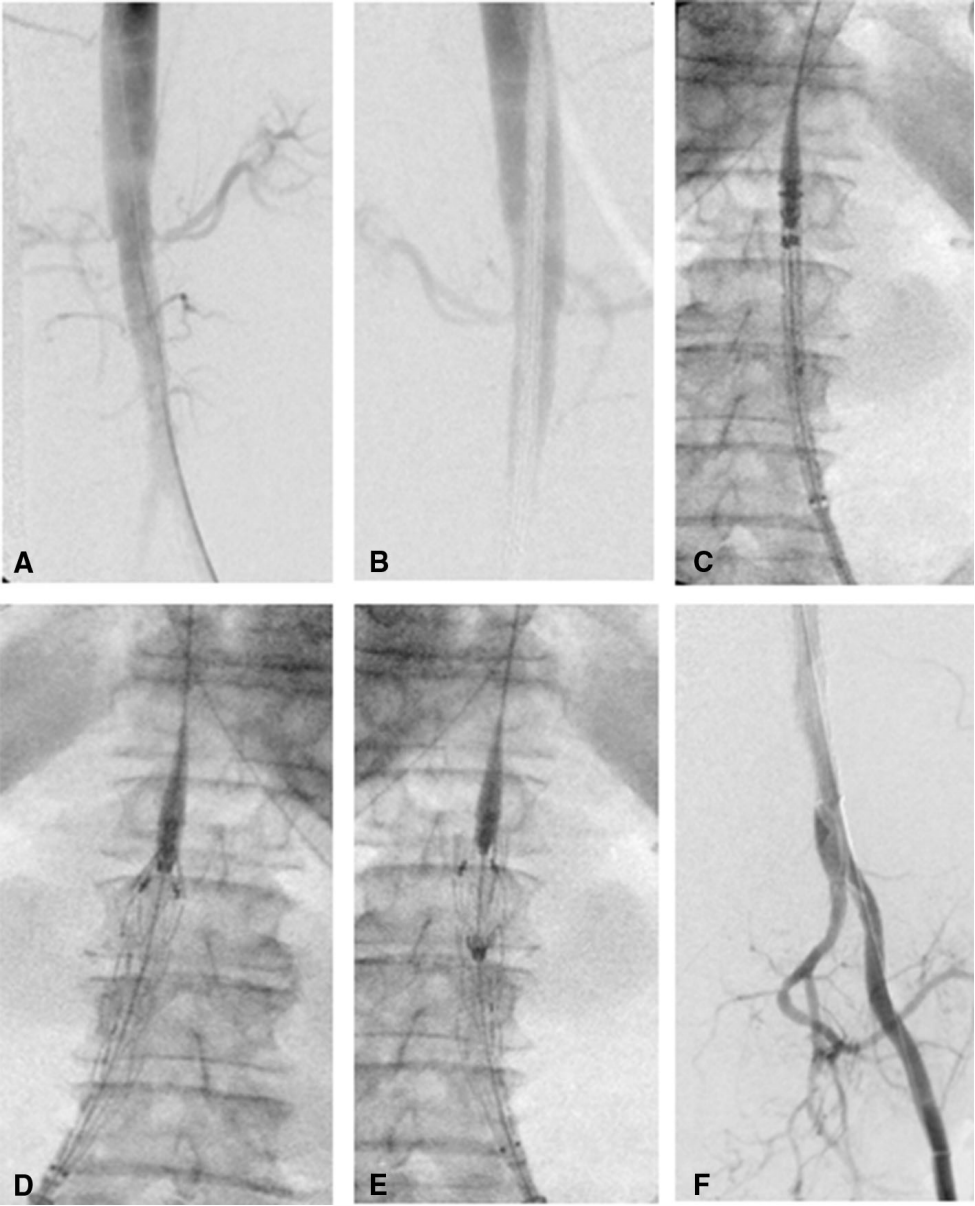
Stent graft intervention to abdominal aorta of Thiel-embalmed human cadaver (thoracic Valiant device and Captivia Delivery System used to demonstrate viability of technique). **A** ipsilateral catheter placement—DSA aortogram [Table 1). Renal arteries evident to plan location of device deployment, **B** DSA Captivia catheter evident, located in aorta to deliver device to infra-renal position, **C** fluoroscopic image of catheter in situ, **D** Valiant stent graft being deployed under fluoroscopic guidance, **E** guide wire remains in device to allow angiographic catheter to be placed in aorta, **F** post-procedure DSA of distal abdominal aorta and left iliac artery which branches distally into internal and external iliac artery. Right iliac occluded by stent graft and therefore not visible on image

A range of different aortic stent graft types were deployed (Table 1): thoracic tube stent graft (Fig. 1), an aorto-uniiliac (Fig. 2) and bifurcated stent graft (Fig. 3). For all interventions, aortic access was secured via the left or right common femoral artery with a 22Fr Sheath (DrySeal sheath, Gore, USA) in situ. A straight stiff guide wire, 0.035 inch diameter × 260 mm long (Amplatz Super Stiff, Boston Scientific), was advanced to the previously planned target location under fluoroscopic guidance. The devices were delivered and deployed according to the manufacturer’s instructions.

**Table 1:** Imaging parameters

Modality	Equipment	Settings	Contrast
X-ray fluoroscopy	OEC Elite 9900 C Arm (GE, USA)	Fluoroscopic imaging 8–12 f.p.s and digital subtraction angiography 25 f.p.s	20-ml Bolus contrast (Omnipaque 300, GE Healthcare, Auckland) was introduced manually
CT	Siemens Biograph mCT scanner (Siemens, Erlangen DE)	Slice thickness 0.6 mm, pitch 0.8, kV 120. Effective mat rotation time, 0.5 s.	Bolus-tracked contrast was initiated manually (Omnipaque 300, GE Healthcare, New Zealand).

### Post-intervention Evaluation

Stent graft placement was evaluated post-procedure by fluoroscopic and DSA angiography. Post-procedure CT was conducted in one cadaver to evaluate device placement, migration and endoleak (Fig. 1).

## Results

**Fig. 4:**
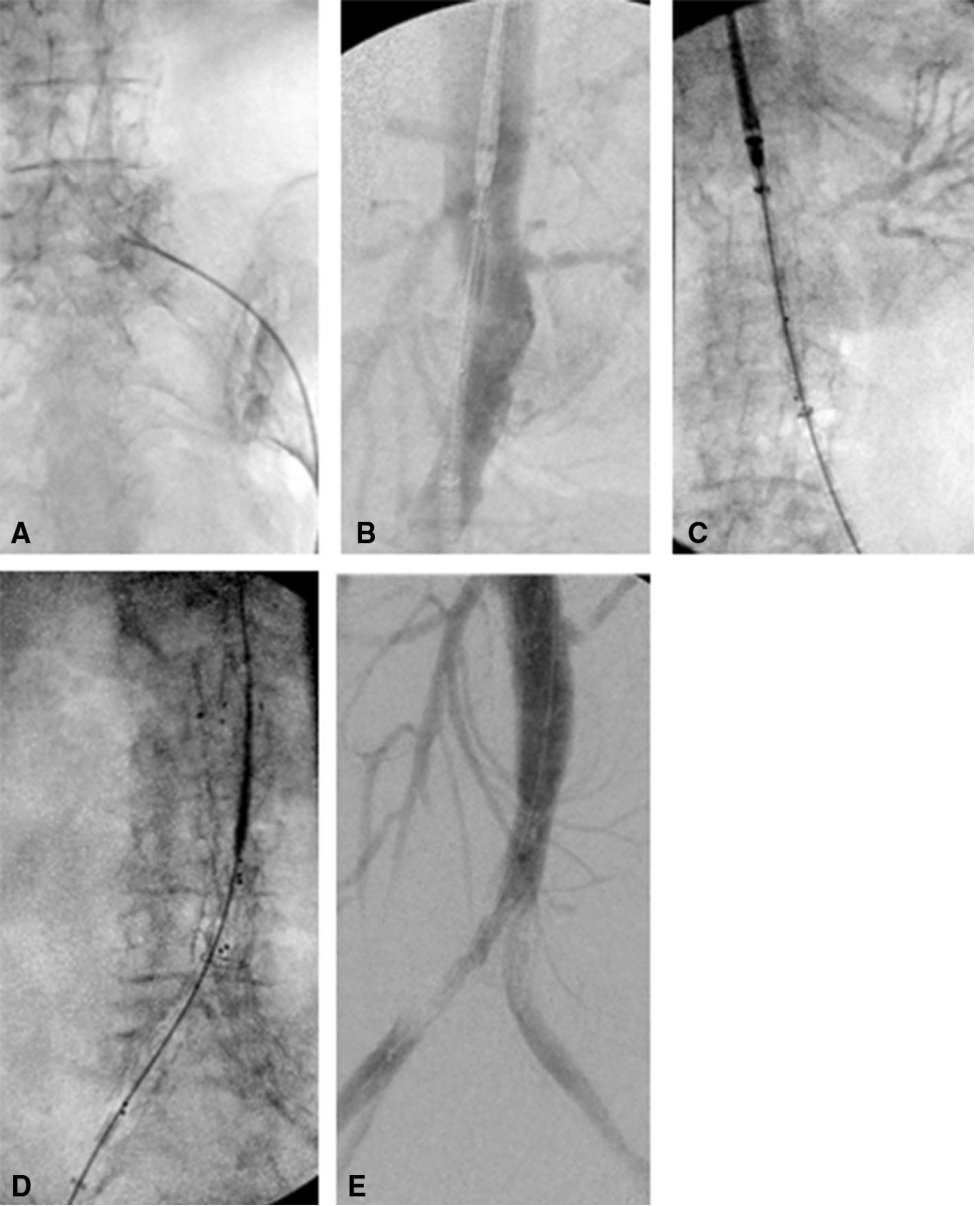
Bifurcating Endurant stent graft intervention to Thiel-embalmed human cadaver. **A** Fluoroscopic image of pigtail angiographic catheter advancing over guide wire from common femoral artery access paint. **B** Pre-deployment digital subtraction angiography showing renal arteries and superior mesenteric artery. **C** Catheter advanced to desired location to locate device in an infra-renal position. **D** Contralateral leg deployment, catheter advanced to dock into device. Leg deployed, **E** post-procedure DSA demonstrating patent device

The simulation followed the pathway similar to live recipients of these devices, with pre-procedure CT providing images to plan the procedure, procedural angiography and post-procedure CT to evaluate the deployed devices (Fig. 4). The arterial landmarks were identified, and the presence and severity of any iliac arterial stenotic disease or tortuosity were noted in all cases. The pre-procedure CT allowed planning of the stent graft procedure and patient-specific co-morbidities into account in the planning simulation, while post-procedure CT allowed assessment of complications such as endovascular leak.

The intravascular temperatures in two cadaveric models were evaluated, with an initial average intraluminal temperature of 16 degrees centigrade which increased to 39.1 degrees centigrade after 4-min perfusion at 2.5 L per min perfusion. Pressures within the aorta were measured during standard perfusion conditions detailed in Table 2.

**Table 2:** Aortic stent grafts used during the procedures

Fig. No.	Device	Dimensions (mm)	Size	Implant location
Figure 1	Valiant thoracic	204 × 30	22Fr	Thoracic aorta
	Valiant thoracic	164 × 30	22Fr	Thoracic aorta
Figure 2	Valiant thoracic	124 × 30	22Fr	Distal abdominal aorta
	Endurant II system	181 × 28		Abdominal aorta—bifurcating to L& R iliac arteries
Figure 3	Endurant II system	9319 to 13		Abdominal aorta—iliac artery

Four aortic stent grafts were successfully deployed according to the procedure plan, in all three cadavers (Figs. 2, 3 and 4). During the procedures, it was noted by the radiologists that the haptic and geometric sensations during the procedure were very similar to that experienced in a live human procedure and superior to that of an animal simulation (85 kg pigs). In particular, the proximal stent positioning, stent deployment, placement of extension pieces, contralateral limb selection and deployment, device retrieval, post-implantation balloon dilatation and angiography check for endoleaks, were found to closely simulate the clinical procedure. Ancillary angioplasty of a coincidental renal artery stenosis and iliac stenosis was also performed with similar favourable results. Post-procedure CT confirmed the precise placement of the TEVAR and EVARS with no evidence of an endoleak (Fig. 1).

## Discussion

Our model is the first to fully perfuse the aorta of a Thiel-embalmed human cadaver by the addition of extracorporeal ante-grade pulsatile flow, originating from the ascending aorta to the entire arterial tree along with temperature and pressure measurement. This approach uniquely provides arterial access from the common femoral arteries and potentially other peripheral vessels to conduct interventions. A great advantage of this model is pulsatile flow within the vessels which allows palpation or ultrasound identification of target vessels followed by standard vascular access technique, providing a closer simulation of the human experience in comparison with animal models, or synthetic and virtual models [10]. Simple virtual training simulators such as Mentice (VIST LAB, Mentice AB, Gothenburg, Sweden) and anatomical simulators with fluoroscopic imaging, such as silicon vascular phantoms (Elstrat, Switzerland), are beneficial tools for gaining basic interventional skills and procedural knowledge but are limited to individual learning and do not provide the complexity of human anatomy, the haptic properties and realism of clinical simulation that the cadaveric model can provide (Table 3).

**Table 3:** Pressure assessment during standard flow conditions (mmHg)

	MAP cadaver 1	MAP cadaver 2
Arch of aorta	38.8	52
Distal aorta	37.5	54
Extracorporeal	78.1	110

The use of Thiel cadavers means a longer “shelf life” with negligible infection hazard compared to fresh frozen cadavers. The application of this Thiel cadaveric perfusion model for endovascular techniques of aortic stent graft placement in the thoracic and abdominal aorta (both aorto-uniiliac and bifurcated grafts) has been shown to be suitable both for device testing and also as an aid to operator training. Particular cadaveric advantages are the range of human anatomy, the presence of complicating co-morbidity such as vessel tortuosity, arterial stenosis and the use of aortic perfusion. In addition, for the purposes of both device testing and operator training, the Thiel cadaveric procedures use “off-the-shelf” standard medical devices (guide wires, catheters, balloons, stent grafts) and standard imaging systems, both fluoroscopic and CT units, imaging contrast media and image acquisition protocols.

In comparison with other published cadaveric models, the Thiel cadaveric model we describe has the capacity to introduce higher volumes of flow at greater velocities due to size of the extracorporeal connection to the aorta. The increased flow rates allow perfused organs such as the kidneys to be fluoroscopically imaged, offering further potential training in interventional techniques as yet to be explored.

Intravascular pressures measured within the cadaveric aorta were lower than normal human intra-aortic pressures. This was expected as peripheral resistance is not present within the cadaver model. Despite the pressure not matching “normal” human in vivo aortic pressure, the flow within the aorta was sufficient to conduct the simulated intervention.

Using Thiel-embalmed cadavers to conduct this type of training limits its application to facilities where these resources and expertise are available. This limitation is balanced by the lack of good *In Vivo* alternatives and therefore offers a specialized solution to the unmet need in this field.

A further limitation of human cadaveric models includes the absence of pathology in predictable and desired locations; in this model, aneurysmal or dissection pathology was absent. However, the cadaveric model did offer vascular pathologies with iliac and renal stenosis being identified, which added to the complexity and relevance of the simulation.

This study demonstrates the Thiel human cadaveric model as a robust, reproducible, high-quality model that is ethically sound, to train multidisciplinary teams in complex endovascular interventions.

## Electronic supplementary material

Below is the link to the electronic supplementary material.

Supplementary material 1 (MOV 5020 kb)

Supplementary material 2 (MOV 1621 kb)

Supplementary material 3 (MOV 1878 kb)
